# Complete Genome Sequence of pAP13, a Large Linear Plasmid of a *Brevibacterium* Strain Isolated from a Saline Lake at 4,200 Meters above Sea Level in Argentina

**DOI:** 10.1128/genomeA.00878-13

**Published:** 2013-11-27

**Authors:** Julian Rafael Dib, Jörg Schuldes, Andrea Thürmer, María E. Farias, Rolf Daniel, Friedhelm Meinhardt

**Affiliations:** Institut für Molekulare Mikrobiologie und Biotechnologie, Westfälische Wilhelms-Universität Münster, Münster, Germanya; PROIMI-CONICET, Tucumán, Argentinab; Genomische & Angewandte Mikrobiologie, Laboratorium für Genomanalyse, Institut für Mikrobiologie und Genetik, Georg-August-Universität, Göttingen, Göttingen, Germanyc

## Abstract

pAP13 is an 89-kb linear plasmid hosted by *Brevibacterium* sp. strain Ap13, an actinobacterium isolated from the feces of a flamingo from an extremely high-altitude lake in Argentina. Because of the ecological importance of the genus *Brevibacterium*, the absolute lack of information concerning *Brevibacterium* linear plasmids, and the possible ecological significance of this unusual plasmid, pAP13 was completely sequenced, including the inversely oriented termini.

## GENOME ANNOUNCEMENT

Since the first description of the genus *Brevibacterium* in 1953 by Breed (1), more than 40 species have been described (http://www.dsmz.de/), including representatives of environmental, biotechnological, and industrial interest, as well as those of clinical significance. Despite such importance, currently only nine sequencing genome projects are running (according to the National Center for Biotechnological Information [NCBI]), and only one complete genome has been reported thus far, for the species *Brevibacterium senegalense* (2).

Among the extrachromosomal elements, several circular plasmids, ranging in size from 4.3 to 70 kb, have been described and characterized (3). In 2010, we showed for the first time the presence of a novel class of extrachromosomal elements in this genus, i.e., the linear plasmid pAP13 (4). Its linearity was demonstrated from pulsed-field gel electrophoresis, and the apparent presence of terminal proteins covalently linked to the 5′-ends was deduced from treatments with exonucleases. The host strain, *Brevibacterium* sp. strain Ap13, which was isolated from the feces of a flamingo in Laguna Aparejos, a high-altitude lake located at approximately 4,200 m above sea level in northwest Argentina, displayed multiple resistance to antibiotics, including the fourth-generation cephalosporin cefepime, which is astonishing since the isolation area is considered to be unspoiled and almost completely devoid of human influences (5).

To check whether pAP13 contributes to the survival of the host bacterium in pristine and extreme environments, the plasmid was completely sequenced.

In total, 71,159 shotgun reads were generated using the 454 GS-FLX system (454 Life Sciences, Roche Applied Science, Branford, CT) for sequencing the entire linear plasmid pAP13. The initial assembly yielded 10 large contigs (>500 bp) determined with the Roche Newbler 1.1 FLX assembler software (454 Life Sciences, Roche Applied Science). In addition, a plasmid library was prepared, and 576 inserts were sequenced. The remaining gaps were closed by PCR-based techniques and Sanger sequencing. Sequence editing was done using GAP4 as part of the Staden software package (6).

In order to obtain the entire plasmid sequence, telomere sequences were achieved by a recently described cloning-independent method (7). In brief, purified plasmid DNA was completely digested with MfeI and BsrGI, which cut close to the 5′- and 3′-ends of the plasmid, respectively, followed by an alkali treatment to remove terminal proteins. The obtained fragments were self-ligated, and finally, a PCR was performed using designed primers to reveal the sequence of the telomeric termini of pAP13.

The nucleotide sequence of pAP13 comprises 89,871 bp, with a G+C content of 62.1%, and contains 113 open reading frames (ORFs), of which 41 ORFs (36%) were assigned to known biological functions.

In the annotation, we found genes involved in the repair of UV-induced DNA damage (UmuC und UmuD proteins), which potentially enhance cell survival in highly irradiated environments, as well as a gene cluster related to amino acids and fatty acid metabolism.

A detailed analysis of the genome and the terminal inverted repeats (TIRs), as well as functional studies, will be the subject of a future publication.

### Nucleotide sequence accession number.

The entire sequence of plasmid pAP13 has been deposited in GenBank under the accession no. KF577590.
